# Sodium, Saturated Fat, and *Trans* Fat Content Per 1,000 Kilocalories: Temporal Trends in Fast-Food Restaurants, United States, 2000–2013

**DOI:** 10.5888/pcd11.140335

**Published:** 2014-12-31

**Authors:** Lorien E. Urban, Susan B. Roberts, Jamie L. Fierstein, Christine E. Gary, Alice H. Lichtenstein

**Affiliations:** Author Affiliations: Lorien E. Urban, Susan B. Roberts, Jean Mayer USDA Human Nutrition Research Center on Aging, Tufts University, Boston, Massachusetts; Jamie L. Fierstein, Christine E. Gary, Freidman School of Nutrition Science and Policy, Tufts University, Boston, Massachusetts.

## Abstract

**Introduction:**

Intakes of sodium, saturated fat, and *trans* fat remain high despite recommendations to limit these nutrients for cardiometabolic risk reduction. A major contributor to intake of these nutrients is foods prepared outside the home, particularly from fast-food restaurants.

**Methods:**

We analyzed the nutrient content of frequently ordered items from 3 US national fast-food chains: fried potatoes (large French fries), cheeseburgers (2-oz and 4-oz), and a grilled chicken sandwich. We used an archival website to obtain data on sodium, saturated fat, and *trans* fat content for these items from 2000 through 2013. The amount of each nutrient per 1,000 kcal was calculated to determine whether there were trends in product reformulation.

**Results:**

Sodium content per 1,000 kcal differed widely among the 3 chains by food item, precluding generalizations across chains. During the 14-year period, sodium content per 1,000 kcal for large French fries remained high for all 3 chains, although the range narrowed from 316–2,000 mg per 1,000 kcal in 2000 to 700–1,420 mg per 1,000 kcal in 2013. Among the items assessed, cheeseburgers were the main contributor of saturated fat, and there was little change in content per 1,000 kcal for this item during the 14-year period. In contrast, there was a sharp decline in saturated and *trans* fat content of large French fries per 1,000 kcal. Post-2009, the major contributor of *trans* fat per 1,000 kcal was cheeseburgers; *trans* fat content of this item remained stable during the 14-year period.

**Conclusion:**

With the exception of French fries, little evidence was found during the 14-year period of product reformulation by restaurants to become more consistent with dietary guidance to reduce intakes of sodium and saturated fat.

## Introduction

High intakes of sodium and of saturated and *trans* fats are associated with increased risk of developing hypertension and cardiometabolic syndrome, respectively (1–3). The 2010* Dietary Guidelines for Americans* indicated that these nutrients are a public health concern as a result of their overconsumption (1). A major contributor to intake of these nutrients is foods prepared outside the home, particularly from fast-food restaurants (4–6), which is concerning because the contribution of foods prepared outside the home has steadily risen during the last 3 decades (7,8).

In a companion article (9), we examined trends over time in the portion size of frequently consumed foods and beverages (3 sizes of fried potatoes [French fries] and regular cola, 2 sizes of cheeseburgers, and a grilled chicken sandwich) (10) among 3 of the top US-based fast-food chain restaurants (11). On the basis of analysis of these data, no consistent temporal trends in portion sizes were found. However, the data indicated that the items assessed contributed a disproportional amount of energy, sodium, saturated fat, and *trans* fat to total daily intake, as assessed by comparing the amounts per serving to recommendations (9).

Independent of issues related to portion size, another potential contributor to excess intake of sodium, saturated fat, and *trans* fat is product formulation, defined here as the amount of these nutrients in a food expressed per unit of energy. Understanding the relationship between portion size and product formulation and the total intake of overconsumed nutrients can aid in development of effective public health strategies designed to encourage people to select among available products for those with the lowest contents of sodium, saturated fat, and *trans* fat. It may also serve to incentivize purveyors to reduce levels in their product formulations.

To address this issue, we summarized the available data for sodium, saturated fat, and *trans* fat content per 1,000 kcal offered by 3 national fast-food chain restaurants for their most frequently ordered menu items: French fries, 2 sizes of cheeseburgers, and a grilled chicken sandwich. Our aim was to document trends in product formulation during a 14-year period, from 2000 through 2013, as a complement to the data on temporal trends in portion sizes provided in the companion article (9).

## Methods

Three fast-food chain restaurants — designated Chain A, Chain B, and Chain C — were selected as examples on the basis of their offering similar menu items, having a national presence, and being among the top 10 for total US sales revenue (11). Chain A was identified as the top restaurant on the basis of sales; the other restaurants were then chosen from the top fast-food restaurants because of their matching menu items. The most commonly ordered menu items offered (10) during the 14-year period included French fries (fried potatoes; large), cheeseburgers (2-oz and 4-oz, uncooked beef weight), and the grilled chicken sandwich (1 available size). Although the companion article (9) included data for regular cola beverages, these data were not included for this analysis, because sodium content varies by local water supply and the beverage does not contain either saturated or *trans* fat. Three sizes of French fries were included in the companion article; however, because their formulation appeared to be similar regardless of size, only data for large-sized French fries were presented. We used the Wayback Machine (www.archive.org/web/web.php) website to collate data. Size designations, when ambiguous according to the chain labels, were determined as described previously (9). From these data, the sodium, saturated fat, and *trans* fat content per 1,000 kcal were calculated.

Time trends were assessed for sodium, saturated fat, and *trans* fat (mg or g per 1,000 kcal) for individual menu items at each chain using simple linear regression models (nutrient component was the dependent variable and year was the independent variable). Differences among chains for individual menu items were assessed using analysis of variance for the mean nutrient components across the 14-year period, and the Tukey post hoc procedure was used to control for multiple comparisons. Statistical analyses were performed using SAS 9.3 (SAS Institute Inc).

## Results

### Sodium

**Figure 1:**
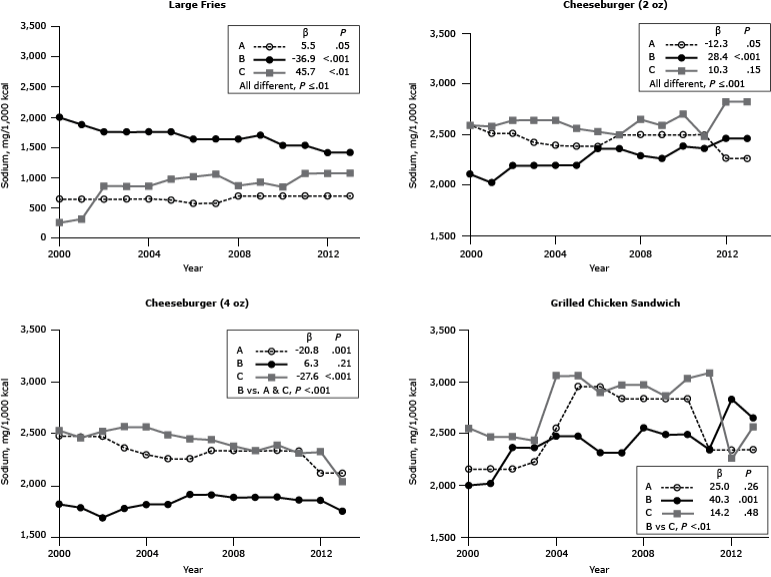
Sodium content (mg/1,000 kcal) for popular menu items at 3 large, national fast-food chains, United States, 2000–2013. Sodium content for large-sized French fries, 2 sizes of cheeseburgers (2-oz and 4-oz), and 1 size of grilled chicken sandwich from chains A, B and C. β estimates and *P* values derived from individual simple linear regression models; chain comparison *P* values derived from ANOVA (analysis of variance) models comparing mean values between restaurants. ^a^ Difference is between Chain B versus Chains A and C. ^b^ Difference is between Chain B versus Chain C. **Table:** 

There was marked heterogeneity in sodium content per 1,000 kcal by food item among the 3 chains (Figure 1). For example, the sodium content per 1,000 kcal of large French fries from Chain B was consistently higher than that of Chains A and C, whereas the sodium content of Chain B’s cheeseburgers was lower than that of Chains A and C.

Chain/Year	Large French Fries	Cheeseburger		Grilled Chicken Sandwich
		2 oz	4 oz	
	Sodium, mg/1,000 kcal			
**Chain A**				
2000	648	2,594	2,472	2,156
2001	648	2,515	2,472	2,156
2002	648	2,515	2,472	2,156
2003	648	2,424	2,358	2,225
2004	654	2,394	2,296	2,550
2005	635	2,387	2,255	2,952
2006	579	2,387	2,255	2,952
2007	579	2,500	2,333	2,833
2008	700	2,500	2,333	2,833
2009	700	2,500	2,333	2,833
2010	700	2,500	2,333	2,833
2011	700	2,500	2,333	2,343
2012	700	2,267	2,115	2,343
2013	700	2,267	2,115	2,343
**β**	5.5	−12.3	−20.8	25.0
***P* Value**	.05	.05	.001	.26
**Chain B**				
2000	2,000	2,111	1,816	2,000
2001	1,880	2,027	1,782	2,018
2002	1,760	2,194	1,682	2,362
2003	1,760	2,194	1,775	2,362
2004	1,760	2,200	1,813	2,474
2005	1,760	2,200	1,813	2,472
2006	1,640	2,364	1,908	2,314
2007	1,640	2,364	1,908	2,314
2008	1,640	2,294	1,883	2,551
2009	1,707	2,265	1,883	2,490
2010	1,537	2,387	1,883	2,490
2011	1,537	2,367	1,855	2,340
2012	1,420	2,464	1,855	2,830
2013	1,420	2,464	1,746	2,647
**β**	−36.9	28.4	6.3	40.3
***P* Value**	<.001	<.001	.21	.001
**Chain C**				
2000	316	2,594	2,531	2,548
2001	319	2,581	2,458	2,467
2002	864	2,645	2,521	2,467
2003	864	2,645	2,563	2,433
2004	864	2,645	2,563	3,056
2005	980	2,563	2,490	3,056
2006	1,019	2,531	2,449	2,892
2007	1,058	2,500	2,440	2,969
2008	873	2,654	2,380	2,969
2009	926	2,593	2,333	2,857
2010	852	2,704	2,389	3,029
2011	1,075	2,481	2,309	3,083
2012	1,075	2,828	2,323	2,256
2013	1,080	2,828	2,033	2,564
**β**	45.7	10.3	−27.6	14.2
***P* Value**	<.01	.15	<.001	.48
**Chain comparison *P* value**	≤.01	≤.001	<.001^a^	<.01^b^

During the 14-year period, the sodium content per 1,000 kcal of Chain A’s large French fries remained stable, that of Chain B’s French fries declined, and that of Chain C’s rose. Different patterns were observed for the cheeseburgers and the grilled chicken sandwich (Figure 1). In 2000, the sodium content per 1,000 kcal of large French fries among chains ranged from 316 mg to 2,000 mg; by 2013, the range narrowed from 700 mg to 1,420 mg per 1,000 kcal. A similar pattern of convergence was observed for the other items assessed, with the exception of the 2-oz cheeseburger.

### Saturated fat

**Figure 2:**
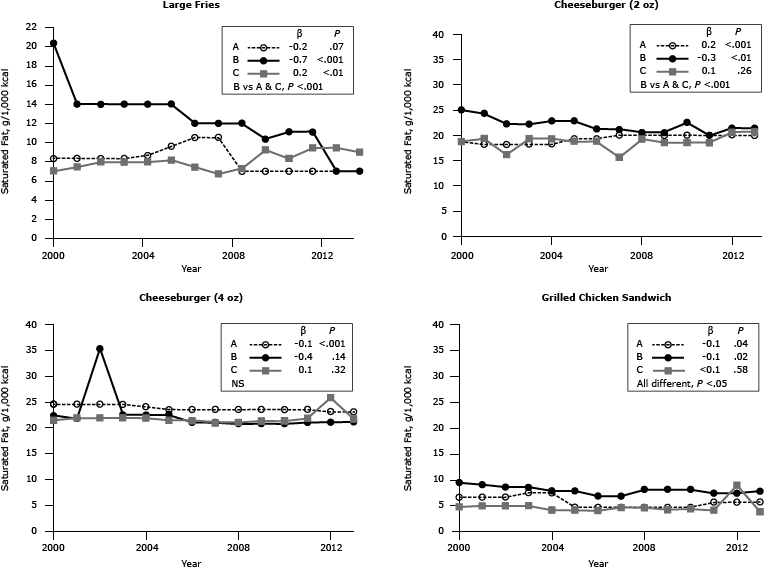
Saturated fat content (g/1,000 kcal) for popular menu items at 3 large, national fast-food chains, United States, 2000–2013. Saturated fat content for large-sized French fries, 2 sizes of cheeseburgers (2-oz and 4-oz), and 1 size of grilled chicken sandwich from chains A, B and C. β estimates and *P* values derived from individual simple linear regression models; chain comparison *P* values derived from ANOVA (analysis of variance) models comparing mean values between restaurants. Abbreviation: NS, nonsignificant. ^a^ Difference is between Chain B versus Chains A and C. **Table:** 

Of the sandwich items assessed, cheeseburgers were the major contributor of saturated fat, with generally about 18 g to 25 g per 1,000 kcal (Figure 2). The saturated fat content of large French fries per 1,000 kcal post-2000 was modest for all chains (range, 6.7–14.0 g). There was a precipitous decline in Chain B’s French fries starting in 2001 and continuing throughout the 14-year period. By 2013, although the range of saturated fat content of French fries per 1,000 kcal was significantly different among chains, in practical terms, the differences were small. For either size of cheeseburger, with few exceptions, there was little change in the saturated fat content per 1,000 kcal between 2000 and 2013. There was a single-year spike in 2002 for Chain B’s large cheeseburger.

Chain/Year	Large French Fries	Cheeseburger		Grilled Chicken Sandwich
		2 oz	4 oz	
	Saturated Fat, g/1,000 kcal			
**Chain A**				
2000	8.3	18.8	24.5	6.7
2001	8.3	18.2	24.5	6.7
2002	8.3	18.2	24.5	6.7
2003	8.3	18.2	24.5	7.5
2004	8.7	18.2	24.1	7.5
2005	9.6	19.4	23.5	4.8
2006	10.5	19.4	23.5	4.8
2007	10.5	20.0	23.5	4.8
2008	7.0	20.0	23.5	4.8
2009	7.0	20.0	23.5	4.8
2010	7.0	20.0	23.5	4.8
2011	7.0	20.0	23.5	5.7
2012	7.0	20.0	23.1	5.7
2013	7.0	20.0	23.1	5.7
**β**	−0.2	0.2	−0.1	−0.1
***P* Value**	.07	<.001	<.001	.04
**Chain B**				
2000	20.3	25.0	22.4	9.4
2001	14.0	24.3	21.8	9.1
2002	14.0	22.2	35.3	8.6
2003	14.0	22.2	22.5	8.6
2004	14.0	22.9	22.5	7.9
2005	14.0	22.9	22.5	7.9
2006	12.0	21.2	21.1	6.9
2007	12.0	21.2	21.1	6.9
2008	12.0	20.6	20.8	8.2
2009	10.3	20.6	20.8	8.2
2010	11.1	22.6	20.8	8.2
2011	11.1	20.0	21.1	7.4
2012	7.0	21.4	21.1	7.5
2013	7.0	21.4	21.1	7.8
**β**	−0.7	−0.3	−0.4	−0.1
***P* Value**	<.001	<.01	.14	.02
**Chain C**				
2000	7.0	18.8	21.4	4.8
2001	7.4	19.4	21.9	5.0
2002	8.0	16.1	21.9	5.0
2003	8.0	19.4	21.9	5.0
2004	8.0	19.4	21.9	4.2
2005	8.2	18.8	21.4	4.2
2006	7.4	18.8	21.4	4.1
2007	6.7	15.6	21.0	4.7
2008	7.3	19.2	21.0	4.7
2009	9.3	18.5	21.3	4.3
2010	8.3	18.5	21.3	4.4
2011	9.4	18.5	21.8	4.2
2012	9.4	20.7	25.8	9.0
2013	9.0	20.7	21.7	3.9
**β**	0.2	0.1	0.1	<0.1
***P* Value**	<.01	.26	.32	.58
**Overall *P* value**	<.001^a^	<.001^a^	NS	<.05

### *Trans* fat

**Figure 3:**
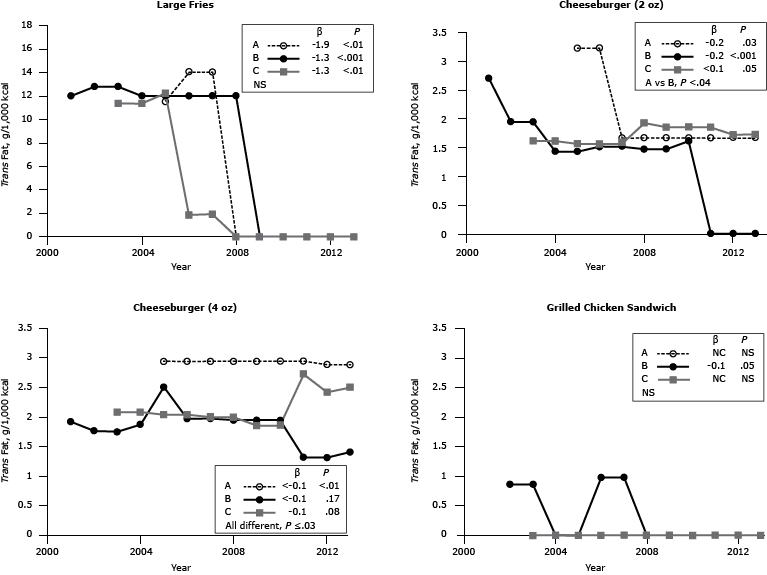
*Trans* fat content (g per 1,000 kcal) for popular menu items at 3 large, national fast-food chains, United States, 2001–2013. *Trans* fat content for large-sized French fries, 2 sizes of cheeseburgers (2-oz and 4-oz), and 1 size of grilled chicken sandwich from chains A, B and C. β estimates and *P* values derived from individual simple linear regression models; chain comparison *P* values derived from ANOVA (analysis of variance) models comparing mean values between restaurants. Abbreviations: —, data not available; NS, nonsignificant. ^a^ Difference is between Chain A and Chain B. **Table:** 

There was evidence of a greater change in reformulation from 2001, when the data for *trans* fat first became available, and from 2013 for *trans* fat than for sodium and saturated fat (Figure 3). After the well-publicized shift from the use of partially hydrogenated fat between 2006 and 2009 (12), the *trans* fat content of large French fries, per 1,000 kcal, declined to undetectable levels (Figure 3). Of the other menu items assessed, the major contributor of *trans* fat was cheeseburgers. Inconsistencies in *trans* fat content were found between sandwiches among chains.

Chain/Year	Large French Fries	Cheeseburger		Grilled Chicken Sandwich
		2 oz	4 oz	
	*Trans* fat, g/1,000 kcal			
**Chain A**				
2001	—	—	—	—
2002	—	—	—	—
2003	—	—	—	—
2004	—	—	—	—
2005	11.5	3.2	2.9	0
2006	14.0	3.2	2.9	0
2007	14.0	1.7	2.9	0
2008	0	1.7	2.9	0
2009	0	1.7	2.9	0
2010	0	1.7	2.9	0
2011	0	1.7	2.9	0
2012	0	1.7	2.9	0
2013	0	1.7	2.9	0
**β**	−1.9	−0.2	<–0.1	NC
***P* Value**	<.01	.03	<.01	NS
**Chain B**				
2001	12.0	2.7	1.9	—
2002	12.8	1.9	1.8	0.9
2003	12.8	1.9	1.8	0.9
2004	12.0	1.4	1.9	0
2005	12.0	1.4	2.5	0
2006	12.0	1.5	2.0	1.0
2007	12.0	1.5	2.0	1.0
2008	12.0	1.5	1.9	0
2009	0	1.5	1.9	0
2010	0	1.6	1.9	0
2011	0	0	1.3	0
2012	0	0	1.3	0
2013	0	0	1.4	0
**β**	−1.3	−0.2	<−0.1	−0.1
***P* Value**	<.001	<.001	.17	.05
**Chain C**				
2001	—	—	—	—
2002	—	—	—	—
2003	11.4	1.6	2.1	0
2004	11.4	1.6	2.1	0
2005	12.2	1.6	2.0	0
2006	1.9	1.6	2.0	0
2007	1.9	1.6	2.0	0
2008	0	1.9	2.0	0
2009	0	1.9	1.9	0
2010	0	1.9	1.9	0
2011	0	1.9	2.7	0
2012	0	1.7	2.4	0
2013	0	1.7	2.5	0
**β**	−1.3	<0.1	0.1	NC
***P* Value**	<.01	.05	.08	NS
**Overall *P* value**	NS	<.04^a^	≤.03	NS

## Discussion

The temporal trends for sodium, saturated fat, and *trans* fat content of French fries, cheeseburgers, and the grilled chicken sandwich were assessed per 1,000 kcal to evaluate potential changes in formulation, independent of portion size. Combining these data with those of portion size will facilitate the refinement of educational programs tailored to address dietary challenges faced by individuals, particularly those at high risk for cardiometabolic disorders, to reduce their intake of overconsumed nutrients. These data may also serve to encourage the food producers to modify food offerings to facilitate compliance with public health recommendations.

The mean sodium intake in the United States is estimated to be 3,400 mg per day (13), which exceeds recommendations for most of the US population (1). Although the 2010 *Dietary Guidelines for Americans *(1) and American Heart Association recommendations (14) advocate reductions in sodium intake, there has been a small increase in absolute intakes during the past 2 decades (15). Despite public health efforts to encourage a reduction in the sodium content of commercially prepared foods (16), the major source of sodium in the US diet (16), there was no consistent temporal pattern — and certainly no clear downward trend, in contrast to findings on *trans* fat content — in the sodium content per 1,000 kcal among the popular menu items in the restaurant chains assessed. In general, differences in the sodium content among chain restaurants were greater when the data were expressed per 1,000 kcal rather than per serving size, as in the companion article (9). This finding suggests that both product formulation and serving size contribute to the differences among chain restaurants in sodium content, with differences in formulation as the major factor. Because the items assessed in this study are those most commonly ordered at fast-food restaurants (9), these items contribute a large amount of sodium to the overconsumption of this nutrient by the US population.

The saturated fat content per 1,000 calories of the items surveyed was consistent between 2000 and 2013, with the exception of a decline in one chain’s fries, which likely occurred due to a change in the frying fat, from beef tallow to partially hydrogenated fat (12). In contrast, per 1,000 kcal, the saturated fat content of cheeseburgers and grilled chicken sandwiches was similar among chains, suggesting that the basic ingredients were similar and that the difference in the saturated fat content of these items among restaurants was attributable primarily to portion size.

When assessed per 1,000 kcal, the *trans* fat content of French fries, independent of fast-food chain, declined in the last decade to become virtually undetectable; this finding confirms the phase-out of partially hydrogenated fat for frying and reflects the success of public health campaigns that target reductions in this nutrient (17–19). The *trans* fat content of the cheeseburgers is likely the result of naturally occurring ruminant fat in the product, making reductions in *trans* fat through reformulation of these menu items feasible only if leaner ground beef and reduced-fat cheese are used. The small inconsistency among chains is likely due to analytical issues such as multiple unsaturated fatty acid isomers containing at least 1 *trans* double bond, suggesting that any differences among chains in *trans* fat content is predominantly due to portion size.

Data on nutrient content per 1,000 kcal should not be considered in isolation, because the total amount of a nutrient contained in an item is determined by both product formulation and portion size. Changing either can contribute to lower intakes, and manipulating both may facilitate the largest change. Accomplishing this may be a challenge, because fast-food restaurants have developed a clientele that expects food to be consistent from visit to visit. Gradual changes may help overcome this challenge (20). One successful approach has been the use of better ingredients for food preparation, such as substituting vegetable oil for partially hydrogenated fat. As the data demonstrate and as has been reported previously, this change has resulted in a reduction in the *trans* fat content of fried items (12). For hamburgers, a shift that could facilitate a reduction in saturated fat would be to use leaner ground beef and reduced-fat cheese.

An alternate approach to making foods from fast-food restaurants more healthful is to introduce new items for which expectations have yet to develop. These new items could give consumers a range of options, which could help them meet dietary recommendations (16) if they consistently varied their choices among these options. Although consumers can lower their intake of overconsumed nutrients by varying the number and choice of items ordered, fast-food restaurants can help by modifying not only the portion size but also the formulation of popular menu items to reduce the supply of these nutrients. To date, reformulation of the items most frequently consumed has not occurred to an adequate extent.
